# Hepatitis C virus prevalence among men who have sex with men: a cross-sectional study in 12 Brazilian cities

**DOI:** 10.1186/s12879-023-08690-2

**Published:** 2023-10-19

**Authors:** Vanessa C. M. Silva, Lígia R. F. S. Kerr, Carl Kendall, Rosa S. Mota, Mark Drew C. Guimarães, Andréa F. Leal, Edgar Merchan-Hamann, Inês Dourado, Maria Amélia Veras, Ana Maria de Brito, Alexandre K. Pontes, Raimunda H. M. Macena, Daniela Knauth, Luana N. G. C. Lima, Socorro Cavalcante, Ana Cláudia Camillo, Ximena P. Díaz-Bermudez, Lisangela C. Oliveira, Laio Magno, Marcílio F. Lemos, Adriana P. Compri, Ana Rita C. Motta-Castro, Regina C. Moreira

**Affiliations:** 1 Laboratório de Hepatites, Centro de Virologia, Instituto Adolfo Lutz, Av. Dr Arnaldo, 355 Pacaembu, São Paulo, SP Brazil; 2 Saúde Comunitária, Faculdade de Medicina, Universidade Federal do Ceará, Fortaleza, CE Brazil; 3 Department of Social, Behavioral and Population Sciences, Tulane University School of Public Health and Tropical Medicine, New Orleans, LA USA; 4 Departamento de Estatística e Matemática Aplicada, Universidade Federal Do Ceará, Fortaleza, CE Brazil; 5 Medicina Preventiva e Social, Universidade Federal de Minas Gerais, Belo Horizonte, MG Brazil; 6 Departamento de Sociologia, Universidade Federal do Rio Grande do Sul, Porto Alegre, RS Brazil; 7 Faculdade de Ciências da Saúde, Saúde Coletiva, Universidade de Brasília, Brasília, DF Brazil; 8 Instituto de Saúde Coletiva, Universidade Federal da Bahia, Salvador, BA Brazil; 9 Departamento de Saúde Coletiva, Faculdade de Ciências Médicas da Santa Casa de São Paulo, São Paulo, SP Brazil; 10 Departamento de Saúde Coletiva, Instituto Aggeu Magalhães, Fiocruz, Recife, PE Brazil; 11 Instituto de Psicologia, Universidade Federal Do Rio de Janeiro, Rio de Janeiro, RJ Brazil; 12 Faculdade de Medicina, Universidade Federal Do Ceará, Fortaleza, CE Brazil; 13 Departamento de Medicina Social, Universidade Federal Do Rio Grande Do Sul, Porto Alegre, RS Brazil; 14 Instituto Evandro Chagas, Ananindeua, PA Brazil; 15 Secretaria de Saúde Do Estado Do Ceará, Fortaleza, CE Brazil; 16 Fundação Alfredo da Matta, Manaus, AM Brazil; 17 Departamento de Saúde Coletiva, Universidade de Brasília, Brasília, DF Brazil; 18 Centro Universitário Autônomo do Brasil (UNIBRASIL), Curitiba, PR Brazil; 19 Departamento de Ciências da Vida, Universidade Do Estado da Bahia (UNEB), Salvador, BA Brazil; 20 Faculdade de Ciências Farmacêuticas, Alimentos e Nutrição, Universidade Federal de Mato Grosso Do Sul, Campo Grande, MS Brazil

**Keywords:** RDS, Sexual and Gender Minorities, Hepatitis C, Brazil

## Abstract

**Background:**

Despite the preventive policies adopted, reduction in sexually transmitted infections (STIs) among men who have sex with men (MSM) has been limited. The risk of hepatitis C virus (HCV) infection has increased among the most vulnerable population groups, including MSM. The aim of this study was to estimate the prevalence of HCV infection and to assess risky practices among MSM from 12 Brazilian cities.

**Methods:**

This study was carried out from June to December 2016 using respondent driven sampling (RDS). Participants completed a self-administered questionnaire to collect behavioral, socioeconomic, and demographic variables. In addition, the rapid diagnostic test (RDT) for HCV was offered. Positive results were sent to Instituto Adolfo Lutz for confirmation.

**Results:**

A total of 4,176 participants were recruited and 23 samples were sent for confirmation. Of these, 16 were confirmed, resulting in a prevalence of 0.7% (95% CI: 0.3%—1.7%). The Southeast region showed a prevalence of 0.9% (95% CI: 0.3—2.6), followed by the South region, with 0.6% (95% CI: 0.2—2.1). The Northeast region had a prevalence of 0.3% (95% CI: 0.1—1.0) and the Midwest 0.1% (95% CI: 0.0—0.7). No positive cases were found in the North. Single men aged 40 years or older were the majority of participants exposed to HCV. High levels of alcohol consumption, illicit drug use, irregular condom use, in addition to infection with other STIs, were associated with exposure to HCV.

**Conclusions:**

STIs continue to be important health problems in Brazil and globally. Many STIs are inapparent for many years until they bring more serious consequences. Extra investment in HCV is also warranted, given that it can be eliminated. Relying solely on clinical data to provide information about inapparent infection, especially in stigmatized populations, will make that goal more difficult to achieve. Surveillance studies, such as the one reported here need to be repeated over time to demonstrate trends and to provide information for evaluation, program and policies. Investments in the most vulnerable populations are critical to achieve the World Health Organization global health goals including the elimination of viral hepatitis by 2030.

## Background

Hepatitis C virus (HCV) infection is an important cause of chronic liver disease globally. A recent important HCV infection literature review and modelling study for the period 2015–2020 estimated that 56.8 million people were infected worldwide at the beginning of 2020 with 12.9 million (23%) diagnosed, and only 641,000 estimated to have initiated treatment in 2020. An estimated 5.5 million deaths (all cause and liver related) are attributed to HCV in the period 2015–2019 [1]. This infection becomes chronic in 75% to 85% of untreated infections. On average, between a third and a quarter of individuals who develop chronic hepatitis C, will develop severe disease if there is no therapeutic intervention [2].

This global public health problem led World Health Organization (WHO) to initiate a campaign to eliminate viral hepatitis by 2030, advocating for reducing new infections of hepatitis B and C by 90% and mortality by 65% [3]. Even given the campaign, the risk of HCV infection has increased among the most vulnerable population groups, including men who have sex with men (MSM) [4].

Brazil adopted measures to reduce the number of new cases and deaths resulting from these infections [3]. To achieve these goals, measures such as early treatment, and mass testing were implemented by the Ministry of Health [2, 4, 5].

Disease prevention and control depends on a complex assessment of the global distribution of HCV infection and determination of associated risk factors. In addition, due to the lack of a vaccine or some form of post-exposure prophylaxis, an effective epidemiological evaluation is required for planning primary prevention actions [6].

Despite the preventive policies adopted, reduction in sexually transmitted infections (STIs) among MSM has been limited, especially among those 25 years of age and younger. This can be observed in several countries, including Brazil [7–9]. Among the factors that explain these differences are risky sexual behaviors, illicit drug abuse, stigma and discrimination, sociodemographic characteristics and barriers to access referral services [10, 11].

As MSM are considered a hidden and hard-to-reach population, special sampling methods are required. In Brazil, respondent-driven sampling (RDS) has been used in two national studies among MSM for HIV since 2006 [12, 13]. RDS is a chain-link referral method that helps access populations without sampling frames that are linked in networks. RDS participants recruit preferably long chains of other participants while reducing the selection bias from initial selected seeds, thus making it possible to derive population estimates from the sample [14–16]. Using RDS among MSM as a surveillance mechanism permits monitoring HCV programs and prevention policies, contributing to an assessment of their effectiveness. Therefore, the purpose of the research presented here was to estimate the prevalence of HCV infection among MSM in Brazil, and to assess risk factors associated with infection in order to improve prevention and treatment and help achieve WHO and Brazilian national goals for elimination.

## Methods

### Study design and population

A cross-sectional observational study was conducted in 12 Brazilian cities, located in the five political-administrative regions of Brazil: the Northern region (Manaus and Belém); the Northeast (Fortaleza, Recife and Salvador); the Midwest (Brasília and Campo Grande); the Southeast (Belo Horizonte, Rio de Janeiro and São Paulo) and the South (Curitiba and Porto Alegre). Participants were recruited from June to December 2016 using RDS, as described in Kendall et al. [17].

The population consisted of men assigned at birth who self-identified as MSM over 18 who had at least one sexual relationship with a man in the last 12 months and who worked, resided or studied in the participating city. Participants who were obviously under the influence of drugs, including alcohol at the time interview, or identified as transgender, were excluded.

Eligible participants who agreed to participate were administered a structured interview using either computer-assisted self-interviewing (CASI) or computer assisted personal interviewing (CAPI) to collect behavioral, socioeconomic and demographic variables. Knowledge of STIs, history of care-seeking for STIs and self-report of STIs, including syphilis and HIV were also collected in the questionnaire.

### Diagnostic tests

Samples were previously screened by rapid diagnostic tests (RDT) (Alere™ HCV, Alere S.A, Brazil) for detection of hepatitis C virus infection among those participants who agreed to test. Patients with positive or indeterminate results were requested to provide a blood sample to confirm the result. After the blood collection, the samples were sent to the hepatitis laboratory of Instituto Adolfo Lutz (IAL) for confirmatory tests by reverse transcription quantitative polymerase chain reaction (RT-qPCR) using the Abbott Real Time HCV™ assay automated system (Abbott Molecular™, Des Plaines, USA) to quantify HCV viral load from the 5´UTR genome region. Samples not detected by RT-qPCR were tested by chemiluminescence (CLIA) using ADVIA Centaur™ XP (Siemens™, Munich, Germany) to evaluate previous exposure and possible false positives in RDT.

Samples testing positive with RT-qPCR were tested by conventional reverse transcription polymerase chain reaction (RT-PCR) for the NS5B genome region following procedures described by Santos et al. [18] for subsequent identification of HCV genotypes and viral sequencing.

### Epidemiological analyzes

During cleanup variables were examined for missing and outliers and adjusted accordingly. In addition, logical consistency analysis was performed.

Gile’s successive sampling (SS) estimator [19] was used to produce weighted estimates using RDS Analyst version 1.7–16. To provide a national estimate, we merged the 12 cities to create a single dataset. We used the Complex Analysis Survey tools in Stata™ 14.0 with each city treated as its own stratum to weight the final results. The social network graphics were drawn for each city, to visualize the distribution of networks chains using NetDraw software [20].

The prevalence of HCV infection was calculated with 95% confidence interval (CI). Univariate logistic models were used in exploratory analyses of the association of variables with the outcome of interest. The strength of the associations was estimated by Prevalence Ratio (PR).

## Results

**Fig. 1:**
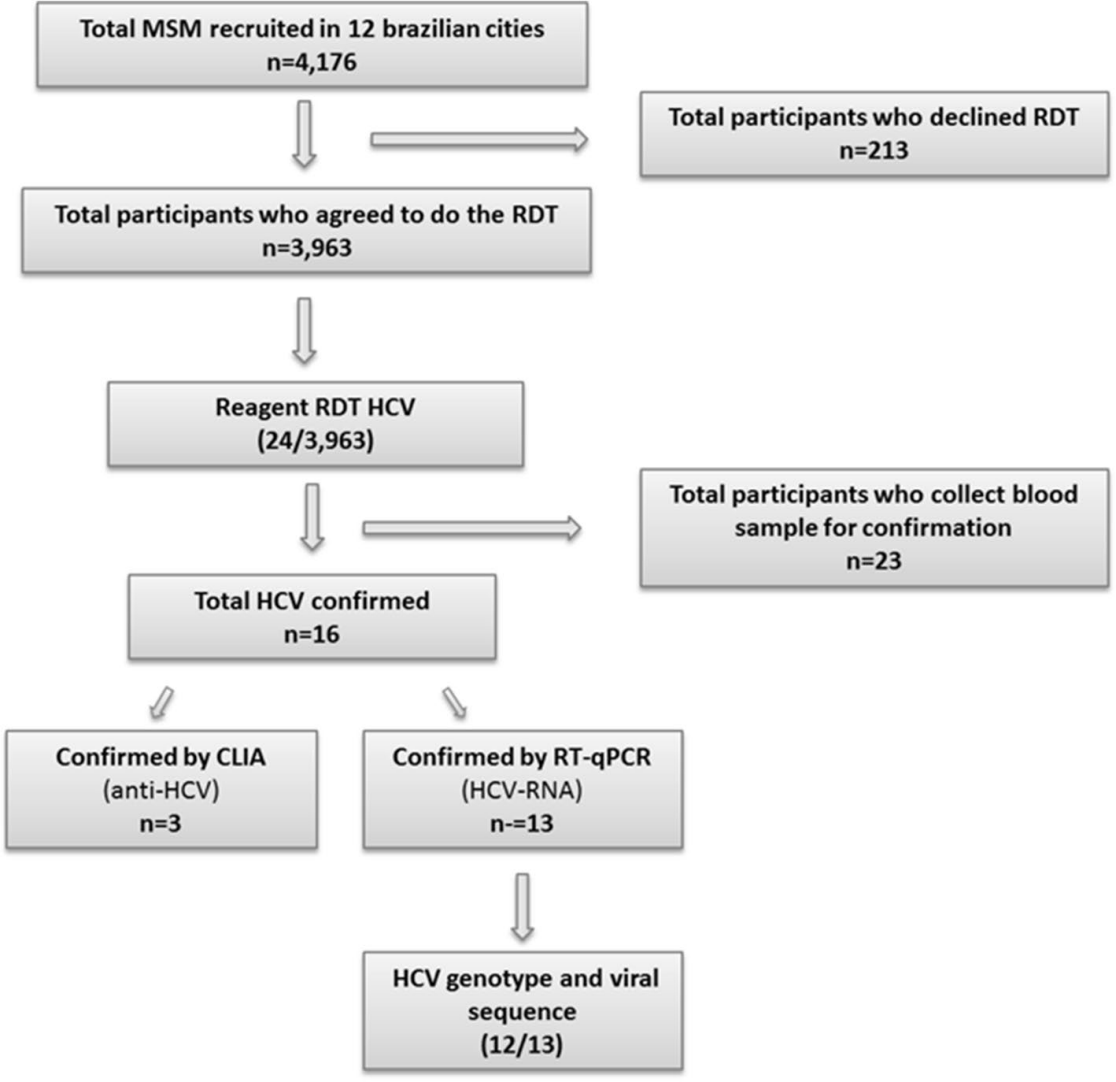
Flowchart of participants’ recruitment and diagnostic tests performed for HCV. *MSM: Men who have sex with men; RDT: Rapid diagnostic tests; anti-HCV: antibodies against Hepatitis C virus; CLIA: Chemiluminescence immunoassay; RT-qPCR: Reverse transcription quantitative polymerase chain reaction

A total of 4,176 MSM were recruited. The characterization of this population was described in Kerr et al. [12] and Kendall et al. [17]. Serological markers (anti-HCV) were tested using RDT in 3,963 blood samples. Of these, 24 were anti-HCV positive, from 9 of the 12 cities studied, and from that, 23 samples were collected and sent to IAL laboratory to confirm the results. One patient refused to provide a blood sample for confirmation (Fig. 1).

Of the 23 samples, 16 were confirmed, either by RT-qPCR or CLIA. This yielded a prevalence for HCV of 0.7% (95% CI: 0.3%-1.7%). The Southeast region showed a prevalence of 0.9% (95% CI: 0.3—2.6), followed by the South region, with 0.6% (95% CI: 0.2—2.1). The Northeast region had a prevalence of 0.3% (95% CI: 0.1—1.0) and the Midwest 0.1% (95% CI: 0.0—0.7). No positive cases were found in the North. The municipalities of São Paulo and Porto Alegre had the highest prevalence, with 1.4% (95% CI: 0.4—4.2) and 1.2% (95% CI: 0.3—4.8), respectively. These data are described in Table 1.

**Table 1:** Estimated prevalence to HCV among MSM, by Brazilian region and city. Analysis of the total positive rapid diagnostic tests compared to confirmatory tests

Brazilian Region/City	HCV RDT Result		HCV confirmed	
	**n/N**^**1**^	**% [CI**^**2**^** 95%]**	**n/N**^**1**^	**% [CI**^**2**^** 95%]**
**Northern**	1/654	0.3 [0.0 – 2.4]	0/654	-
Manaus	1/351	0.6 [0.1 – 3.8]	0/351	-
Belém	0/303	-	0/303	-
**Northeast**	**10/1026**	**0.9 [0.5 – 1.9]**	**5/1025**	**0.3 [0.1 – 1.0]**
Fortaleza	4/338	1.3 [0.4 – 3.6]	1/338	0,1 [0.0 – 0.9]
Recife	3/346	1.4 [0.4 – 4.5]	1/345	0.6 [0.1 – 4.1]
Salvador	3/342	0.4 [0.1 – 1.6]	3/342	0.4 [0.1 – 1.6]
**Midwest**	**1/707**	**0.1 [0.0 – 0.7]**	**1/707**	**0.1 [0.0 – 0.7]**
Campo Grande	1/351	0.4 [0.1 – 2.9]	1/351	0.4 [0.1 – 2.9]
Brasília	0/356	-	0/356	-
**Southeast**	**8/924**	**1.0 [0.4 – 2.6]**	**7/924**	**0.9 [0.3 – 2.6]**
Belo Horizonte	0/330	-	0/330	-
São Paulo	6/338	1.6 [0.6 – 4.3]	5/338	1.4 [0.4 – 4.2]
Rio de Janeiro	2/256	0.3 [0.1 – 1.5]	2/256	0.3 [0.1 – 1.5]
**South**	**4/652**	**0.7 [0.2 – 2.1]**	**3/652**	**0.6 [0.2 – 2.1]**
Curitiba	1/335	0.2 [0.0 – 1.5]	1/335	0.2 [0.0 – 1.5]
Porto Alegre	3/317	1.4 [0.4 – 4.8]	2/317	1.2 [0.3 – 4.8]
**Total**	**24/3963**	**0.9 [0.4 – 1.8]**	**16/3962**	**0.7 [0.3 – 1.7]**

*n* Total of positive HCV RDT/ HCV corfirmed results; *N* Total analyzed samples; *RDT* Rapid diagnostic test; 1: Observed outcomes; 2: Weighted outcomes, *CI *Confidence interval, *HCV *Hepatitis C virus

The presence of HCV RNA was detected in 81.2% (13/16) anti-HCV positive samples. Of these, the amplification of HCV RNA by conventional RT-PCR. Amplification could not be conducted in one sample due to low viral load. Genotyping by sequencing was performed for these 12 samples. The predominant HCV genotype was 1a, detected in 41.7% (5/12), followed by genotypes 3a, 33.3% (4/12) and 1b, with 25% (3/12) (Table 2).

**Table 2:** Comparison of positive tests for hepatitis C by city

Cities	HCV RDT positive	RT-qPCR	Log	CLIA	Genotype	False positive
**Manaus**	515	ND	ND	NR	NP	**1**
**Fortaleza**	286	D	6.09	NP	3a	**3**
	363	ND	ND	NR	NP	
	1310	ND	ND	NR	NP	
	1411	ND	ND	NR	NP	
**Recife**	08	D	6.71	NP	3a	**1**
	1340	ND	ND	NR	NP	
	NS	NP	NP	NP	NP	
**Salvador**	04	D	6.56	NP	3a	**0**
	1027	D	5.03	NP	1b	
	1084	D	6.34	NP	1a	
**Campo Grande**	1030	D	3.23	NP	NP	**0**
**São Paulo**	25	D	5.89	NP	1a	**1**
	28	D	6.10	NP	1b	
	199	ND	ND	NR	NP	
	1121	D	6.59	NP	1a	
	1241	D	5.77	NP	1a	
	1249	D	6.06	NP	3a	
**Rio de Janeiro**	239	ND	ND	R	NP	**0**
	949	ND	ND	R	NP	
**Curitiba**	841	D	7.22	NP	1a	**0**
**Porto Alegre**	793	ND	ND	NR	NP	**1**
	866	ND	ND	R	NP	
	1030	D	5.47	NP	1b	
**TOTAL**	**23**	**13**	**13**	**3**	**12**	**7**

*RDT* Rapid diagnostic tests, *D* Detected, *ND* Not detected, *NS* Not sent, *NP* Not performed, *R* Reagent, *NR* Not reagent, *CLIA* Chemiluminescence immunoassay, *RT-qPCR* Reverse transcription quantitative polymerase chain reaction, *HCV* Hepatitis C virus

**Fig. 2:**
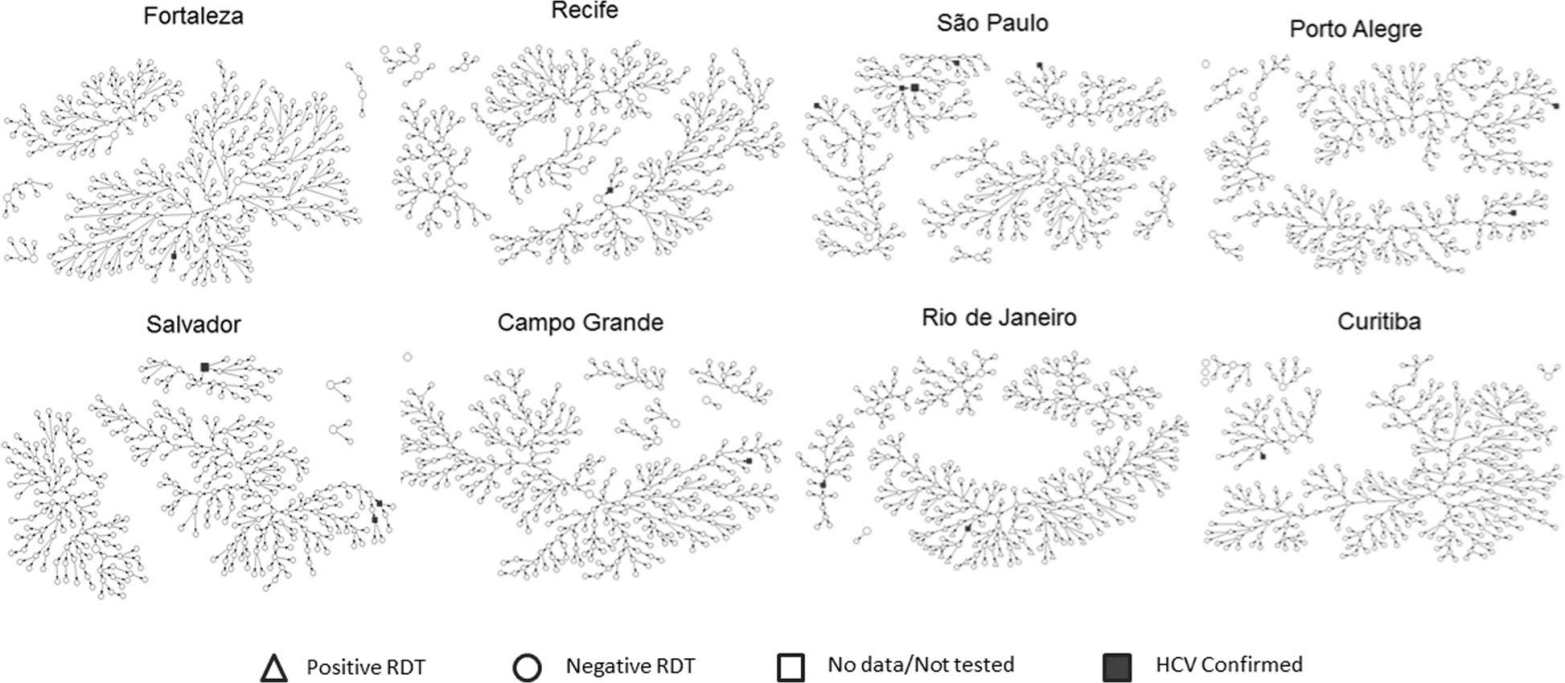
Recruitment networks established by 4,176 MSM in the 12 Brazilian cities, 2016. *RDT: Rapid diagnostic tests; HCV: Hepatitis C virus

Statistical analysis was conducted with weighted values ​​and prevalence ratio (PR) analysis, since the prevalence of HCV in this population was low. Recruitment networks trees were constructed by city (Fig. 2). The length of the longest chain ranged from 8 waves in Belém to 21 waves in Curitiba. The larger figures (squares/circles/triangles) represent the seeds and the smaller figures represent the social network demonstrated by RDS recruitment. The presence of large major components provide some confidence that the values reported are for the population as a whole rather than the characteristics of the seeds.

Table 3 presents the results of the analysis of the questionnaire data. Of the characteristics presented, white race, middle-class status, age > 40 years, single civil status, and low level of education are most associated with a positive HCV status.

**Table 3:** Socioeconomic and demographic characteristics of participants in 12 Brazilian cities, 2016

**Characteristics**	**HCV positive**		**HCV negative**		
	**n**^**1**^**/N**^**1**^	**%**^**2**^** [CI**^**2**^** 95%]**	**n**^**1**^**/N**^**1**^	**%**^**2**^** [CI**^**2**^** 95%]**	**PR**^**2**^** [CI**^**2**^** 95%]**
**Age (years)**					
< 30 years	3/3056	0.20 [0.10 – 0.90]	3053/3056	99.80 [99.10 – 99,90]	1.000
30 a 40 years	2/559	0.30 [0.10 – 1.70]	557/559	99.70 [98.30 – 99.90]	1.403 [0.178 – 11.084]
> 40 years	11/309	1.34 [0.47 – 3.74]	1513/309	96.30 [87.50 – 99.00]	15.038 [2.462 – 91.857]
**Socioeconomic Strata (ABEP)**^a^					
A/B (Higher)	7/1813	0.54 [0.19 – 1.50]	1806/1813	99.46 [98.50 – 99.81]	2.651 [0.551 – 12.740]
C (Midlle)	5/1569	0.79 [0.14 – 4.27]	1564/1569	99.21 [95,73 – 99.86]	3.910 [2.462 – 91.857]
D/E (Lower)	3/536	0.20 [0.06 – 0.66]	533/536	99.80 [99,34 – 99.94]	1.000
**Civil status**					
Married/stable union	2/509	0.12 [0.03 – 0.50]	507/509	99.88 [99.50 – 99.97]	1.000
Single/separate/widower	13/3398	0.67 [0.23 – 1.88]	3385/3398	99.33 [98.12 – 99.77]	5.744 [0.958 – 34.443]
**Years of school**					
≤ 4 years	5/344	2.95 [0.55 – 14.35]	339/344	97.05 [85.65 – 99.45]	11.072 [ -]
5–11 years	0/603	-	603/603	-	- [ -]
High school/incomplete college	7/2527	0.27 [0.08 – 0.85]	2520/2527	99.73 [99,15 – 99.92]	1.000
College graduate	4/453	1.46 [0.42 – 4.89]	449/453	98.54 [95,11 – 99.58]	5.479 [ -]
**Skin color**					
White	7/1234	1.23 [0.31 – 4.83]	1227/1234	98.77 [95.17 – 99.69]	12.639 [2.183 – 73.000]
Black	4/852	0.65 [0.18 – 2.36]	848/852	99.35 [97.64 – 99.82]	6.641 [1.221 – 36.026]
Mixed/brown	4/1671	0.10 [0.03 – 0.29]	1667/1671	99.90 [99.71 – 99.97]	1.000

^a^*ABEP* Brazilian Association of Research Companies, *n* Total of positive/negative results, *N* Total analyzed, *1* Observed outcomes, *2* Weighted outcomes, *CI* Confidence interval, *PR* Prevalence ratio, *HCV* Hepatitis C virus

Regarding socio-behavioral characteristics and risk practices, an association was observed between HCV infection and high levels of alcohol consumption, use of illicit drugs, and irregular condom use. In addition, reports of previous STIs such as syphilis and/or HIV were associated with HCV. These data are reported in Table 4.

**Table 4:** Socio-behavioral and risk practices characteristics of participants in 12 Brazilian cities, 2016

**Characteristics**	**HCV positive**		**HCV negative**		
	**n**^**1**^**/N**^**1**^	**%**^**2**^** [CI**^**2**^** 95%]**	**n**^**1**^**/N**^**1**^	**%**^**2**^** [CI**^**2**^** 95%]**	**PR**^**2**^** [CI**^**2**^** 95%]**
**Alcohol consumption levels**					
Low-risk drinking or abstaining	7/1952	0.31 [0.09 – 1.01]	1945/1952	99.69 [98.99 – 99.91]	1.000
Risk consumption/probable dependence	8/1816	0.94 [0.25 – 3.43]	1808/1816	99.06 [96.57 – 99.75]	3.062 [0.520 – 18.027]
**Has used illicit drugs in the last 6 months?**					
Yes	6/1914	0.82 [0.20 – 3.22]	1908/1914	99.18 [96.78 – 99.80]	2.198 [0.385 – 12.538]
No	9/1934	0.37 [0.13 – 1.06]	1925/1934	99.63 [98.94 – 99.87]	1.000
**Has sniffed/injected cocaine in the last 6 months?**					
Yes	3/627	1.53 [0.25 – 8.73]	624/627	98.47 [91.27 – 99.75]	4.207 [0.574 – 30.838]
No	12/3209	0.36 [0.15 – 0.88]	3197/3209	99.64 [99.12 – 99.85]	1.000
**Age of first sexual intercourse**					
≤ 14 anos	11/1648	0.67 [0.26 – 1.72]	1637/1648	99.33 [98.28 – 99.74]	1.236 [0.186 – 8.231]
≥ 15 anos	4/2218	0.55 [0.10 – 2.79]	2214/2218	99.45 [97.21 – 99.90]	1.000
**Used condoms in first sexual intercourse**					
No/ Do not know	12/2203	0.89 [0.27 – 2.84]	2191/2203	99.11 [97.16 – 99.73]	3.978 [0.509 – 31.061]
Yes	3/1682	0.22 [0.04 – 1.20]	1679/1682	99.78 [98.80 – 99.96]	1.000
**Have you ever had symptoms of STI?**					
Yes	4/723	1.96 [0.45 – 8.19]	719/723	98.04 [91.81 – 99.55]	17.365 [3.365 – 89.605]
No	11/3179	0.26 [0.10 – 0.65]	3168/3179	99.74 [99.35 – 99.90]	1.000
**Has a doctor ever told you that you ever had an STI?**					
Yes	10/1009	1.86 [0.58 – 5.73]	999/1009	98.14 [94.27 – 99.42]	55.360 [10.645 – 286.538]
No	5/2862	0.11 [0.03 – 0.35]	2857/2862	99.89 [99.65 – 99.97]	73.185 [9.460 – 564.626]
**Syphilis**					1.000
Active infection	10/534	2.08 [0.84 – 5.10]	524/534	97.92 [94.90 – 99.16]	
Scar	3/479	2.76 [0.59 – 11.87]	476/479	97.24 [88.13 – 99.41]	18.153 [4.792 – 68.766]
Negative	3/2891	0.04 [0.01 – 0.15]	2888/2891	99.96 [99.85 – 99.99]	1.000
**HIV**					
Positive	6/535	1.38 [0.49 – 3.84]	529/535	98.62 [96.16 – 99.51]	1.000
Negative	7/3389	0.08 [0.03 – 0.18]	3382/3389	99.92 [99.82 – 99.97]	3.062 [0.520 – 18.027]

n: Total of positive/negative results; N: Total analyzed; 1: Observed outcomes; 2: Weighted outcomes; *CI* Confidence interval, *PR* Prevalence ratio, *STI* Sexually transmitted infections, *HCV* Hepatitis C virus, *HIV* Human immunodeficiency virus

Four of the 16 participants with HCV reported being aware of the infection, 2 reported having a previous negative test result and 10 participants reported not previously testing or knowing if they had been tested.

## Discussion

WHO and member states has proposed a plan to eliminate viral hepatitis B and C by 2030. These hepatitides are responsible for 96% of all mortality caused by viral hepatitis. To monitor progress toward this goal, information about prevalence of HCV is required [3]. As is shown in our study, most HCV positive individuals do not know of their status, nor have they been tested in clinical encounters. This demonstrates the need for special studies, such as the one reported here, to capture information about prevalence in high-risk populations.

The prevalence of HCV observed in this population was similar to other Brazilian HCV studies on MSM using RDS [21, 22]. Brazil is a country with a low prevalence of HCV [23]. Although the study population reported high-risk practices that could be related to transmission, HCV infection was not more frequent in MSM than in the general Brazilian population [24]. Considering the population-based study carried out in Brazilian capitals between 2005 and 2009 [24], the prevalence observed for HCV in the present study was lower. On the other hand, studies conducted around the world showed higher rates [25–27].

These differences may be associated with hepatitis targeted public health efforts aimed at controlling this infection, as well as other STIs, in vulnerable populations. Furthermore, mandatory HCV screening in blood banks, in addition to the increase in the offer of treatment and diagnosis, may also be associated with a decrease in prevalence in this population [5, 28]. Finally, injection drug use in Brazil is low, according to the third national survey on drug use in the Brazilian population published in 2017 [29]. Prevention and harm reduction programs, especially those delivered through LGBT supportive non-governmental organizations (NGOs), may help achieve WHO and national goals [28].

The genotypes found (GT1 and GT3) corroborate Brazilian estimates [30, 31]. The identification of risk factors as well as investigation of transmission between individuals benefit from genotyping [32]. The distribution of genotypes may vary according to geographic location, and monitoring this distribution is important in defining epidemiological trends, introducing new genotypes, and determining associated transmission routes [33, 34].

We observed some false positives in the RDTs. Screening tests are not definitive, but they do ehance access to more definitive tests. Screening tests should have high sensitivity to ensure that all true positives are found in the tested population. False positives may occur more frequently due to this high sensitivity [35, 36] and the Brazilian Ministry of Health recommends that after RDT, the RT-qPCR test be carried out as a complementary test [2]. This confirmation assists in appropriate treatment and allows monitoring by specialized professionals. On the other hand, confirming the false positive result avoids unnecessary treatment and psychological damage.

Some observed characteristics were related to an increased probability of contracting HCV infection. Among positive HCV individuals, age 40 years or older was an important factor, probably due to the increased risk of exposure over time. Injuries to the anorectal mucosa caused by anal intercourse can also increase the risk of transmission of HCV and other STIs [37].

The prevalence of HCV is observed in older age groups in several studies and they may be up to five times more likely to be infected than other groups [38]. This fact may be related to risk during transfusions, infected medical equipment, or procedures adopted before precautions established for the control of this infection were implemented or even before the discovery of HCV and the development of diagnostic methods. In Brazil, epidemiological studies also show a higher prevalence in older age groups [31].

Another point observed was the high number of coinfections among HCV positive individuals. Syphilis and/or HIV were observed in most of these participants. This fact can be explained by the high exposure to STIs due to risky sexual practices such as unprotected sexual intercourse. It is known that the presence of other STIs can facilitate infections by other diseases, including HCV [39]. Intrarectal seminal deposition is considered the main form of sexual transmission of HCV among MSM [40]. Sexual transmission can be associated with reported decline in regular condom use that may be associated with successful HIV treatment and the use of pre-exposure prophylaxis. While these programs are altogether laudatory, they need to be accompanied by new condom promotion interventions to prevent HCV infection in this population [41].

Most study participants reported high-risk behaviors, including those with evidence of active HCV infection. In addition, 10 HCV-positive participants also had STI coinfection, which could be a source of transmission not only of HCV, but other STIs. Several studies highlight important gaps in prevention, including a decline in the promotion of condom use and other forms of preventive behavior [12, 42].

This study contributed to the diagnosis of the participants in this research who were quickly referred for treatment, preventing the progression of the disease, in addition to preventing the spread of HCV. Of the 16 positive participants, 12 were diagnosed through this research, highlighting another contribution of surveillance to public health.

This research, as all research, has limitations. The fact that it is a cross-sectional study does not allow a causal relationship between the associated factors to be established. In addition, despite the use of the CASI system, self-report is problematic, especially for sensitive and intimate behaviors [17]. The limitations of RDS are documented; its use requires accepting several strong assumptions, and remains controversial [43, 44]. However, its applications to surveillance and research continue and theoretical and operational efforts continue to respond to the threats of bias and to improve statistical estimates [45–47].

The study of social networks in the context of STIs is an indispensable research and intervention tool since transmission can quickly occur in sexual relationship networks. For MSM exploring these contact networks is essential since general population surveys are unlikely to identify the full range of MSM due to stigma and discrimination [48, 49]. Use of RDS for intervention is generally underappreciated although RDS began as an intervention delivery tool [50].

People who inject drugs, sex workers, transgender individuals, as well as MSM, are the most important populations in concentrated epidemics. As there is no gold standard reference framework for probabilistic sampling, our choices are restricted to RDS and time-location sampling, which both suffer from bias [48]. On the other hand, knowing these populations as well as we can is essential to guide prevention and treatment programs [28].

Nevertheless, RDS is considered one of the only effective methods of recruiting hard-to-reach vulnerable individuals, and operational features and data collection instruments are well defined [43, 46]. The present study followed the guidelines of the Report of Observational Studies in Epidemiology for RDS (STROBE-RDS) [51].

## Conclusions

STIs, continue to be important health problems in Brazil and globally. Many STIs are inapparent for many years until they bring more serious consequences. Extra investment in HCV is also warranted, given that it can be eliminated. Relying solely on clinical data to provide information about inapparent infection, especially in stigmatized populations, will make that goal more difficult to achieve. Surveillance studies, such as the one reported here need to be repeated over time to demonstrate trends and to provide information for evaluation, program and policies. Investments in the most vulnerable populations are critical to achieve the World Health Organization (WHO) global health goals including the elimination of viral hepatitis by 2030.

## Data Availability

The datasets used and/or analyzed during the current study are available from the corresponding author on reasonable request.
